# Association of excessive smartphone use with psychological well-being among university students in Chiang Mai, Thailand

**DOI:** 10.1371/journal.pone.0210294

**Published:** 2019-01-07

**Authors:** Arunrat Tangmunkongvorakul, Patou Masika Musumari, Kulvadee Thongpibul, Kriengkrai Srithanaviboonchai, Teeranee Techasrivichien, S. Pilar Suguimoto, Masako Ono-Kihara, Masahiro Kihara

**Affiliations:** 1 Research Institute for Health Sciences, Chiang Mai University, Sriphum, Muang Chiang Mai, Thailand; 2 Department of Global Health and Socio-epidemiology, Kyoto University School of Public Health, Graduate School of Medicine Yoshida-Konoe-cho, Sakyo-ku, Kyoto, Japan; 3 Department of Psychology, Faculty of Humanities, Chiang Mai University, Muang District, Chiang Mai, Thailand; 4 Department of Community Medicine, Faculty of Medicine, Chiang Mai University, Chiang Mai, Thailand; 5 Center for Medical Education, Graduate School of Medicine, Kyoto University, Yoshida-Konoe-cho, Sakyo-ku, Kyoto, Japan; Chiba Daigaku, JAPAN

## Abstract

**Background:**

Despite the pervasive use of smartphones among university students, there is still a dearth of research examining the association between smartphone use and psychological well-being among this population. The current study addresses this research gap by investigating the relationship between smartphone use and psychological well-being among university students in Thailand.

**Methods:**

This cross-sectional study was conducted from January to March 2018 among university students aged 18–24 years from the largest university in Chiang Mai, Thailand. The primary outcome was psychological well-being, and was assessed using the Flourishing Scale. Smartphone use, the primary independent variable, was measured by five items which had been adapted from the eight-item Young Diagnostic Questionnaire for Internet Addiction. All scores above the median value were defined as being indicative of excessive smartphone use.

**Results:**

Out of the 800 respondents, 405 (50.6%) were women. In all, 366 (45.8%) students were categorized as being excessive users of smartphones. Students with excessive use of smartphones had lower scores the psychological well-being than those who did not use smartphone excessively (*B* = -1.60; *P* < 0.001). Female students had scores for psychological well-being that were, on average, 1.24 points higher than the scores of male students (*P* < 0.001).

**Conclusion:**

This study provides some of the first insights into the negative association between excessive smartphone use and the psychological well-being of university students. Strategies designed to promote healthy smartphone use could positively impact the psychological well-being of students.

## Introduction

Smartphones have become ubiquitous, with growing adoption rates across low-, middle, and high-income countries [1]. The term smartphone generally refers to a mobile phone offering some computer-like functionalities [2]. These advanced functionalities and applications, including Internet capability, portability, and accessibility have made smartphones essential equipment for many in people in their everyday lives.

There is a growing amount of literature which indicates that many people overuse their phones in ways which interfere with their daily lives and mental well-being [3, 4]. Various terms have been used to describe different patterns of smartphone overuse. These include for example “excessive smartphone use”, “problematic smartphone use”, and “smartphone addiction” [5, 6]. For the sake of simplicity, we have adopted the term “excessive smartphone use” in this manuscript to refer to the complete range of problem smartphone use patterns. Excessive smartphone use is associated with a range of negative social and health outcomes, such as poor academic and work performance [7, 8], reduced in-person social interaction [9, 10], traffic road accidents [11], and mental health problems (depression and anxiety) [5, 12].

The smartphone adoption rate has been particularly high among university students. Many studies have shown that young people tend to adopt electronic devices earlier, as compared to other demographic groups [13]. University students also face unique circumstances (e.g., academic stress; the anxiety about planning their future careers; and having their first experiences with independent living) which tend to threaten their mental health and well-being [14–16]. There is a growing body of evidence, which has been comprehensively compiled in a recent systematic review [5], showing that excessive smartphone use is associated with depression, anxiety, and stress among university students in diverse settings. However, there is a dearth of academic research on the link between excessive smartphone use and psychological well-being of university students. In contrast to subjective well-being, which falls within the hedonic perspective on well-being (happiness, life-satisfaction, self-esteem, positive affect etc.) [17], psychological well-being represents the core aspects of optimal human functioning. Psychological well-being is based on the eudaimonic perspective on well-being, and emphasizes aspects of psychological functioning such as social contribution, positive relationship with others, personal growth, self-acceptance, and purpose in life [18–20]. Traditionally, most research has focused on the subjective form of well-being, however, there has been growing attention on psychological form of well-being in the past years [21, 22].

To date, we have found only one study investigating the link between smartphone addiction and psychological well-being (eudaimonic facet of well-being) [23], conducting among university students in Turkey. It showed that smartphone addiction was associated with lower scores of psychological well-being, however, the association was derived from a bivariate analysis, and there was no control for some important variables [23]. The current study examines the relationship between smartphone use and psychological well-being among university students in Chiang Mai, Thailand.

## Methods

### Study design, participants and setting

This is a cross-sectional study conducted from January to March 2018, among university students aged 18–24 years in Chiang Mai, Thailand. The students were recruited from Chiang Mai University, the first and largest university in Northern Thailand. The sample size was calculated based on Krejcie and Morgan’s formula [24]. The overall sample was conceptualized as comprising two subsamples, which represented males and females separately. The required sample size was calculated to be 395 for male students and 405 for female students, giving a total sample size of 800. The recruitment process consisted of the following steps. Chiang Mai University consists of twenty faculties and one college. The recruitment process included the following steps. Firstly, we grouped the faculties into three main areas: (a) Health Sciences (six faculties); (b) Science and Technology (five faculties and one college); and (c) Humanities and Social Sciences (nine faculties). Secondly, we randomly selected four faculties from each of the main areas. A total of twelve faculties were selected: i) the Health Science area (the Faculties of Medicine, Dentistry, Pharmacy, and Associated Medical Sciences); ii) the Science and Technology area (the Faculties of Engineering, Architecture, Science, and Agro-Industry); and iii) the Humanities and Social Sciences area (the Faculties of Social Science, Education, Economics, and Political Science and Public Administration). The years of study were 1 to 6^th^ year for the faculties of Medicine, Dentistry and Pharmacy, and 1 to 4^th^ year for the rest of the faculties. Thirdly, the selected faculties were stratified by year of study (four groups: 1^st^ year; second year; third year; and fourth year and over) and by gender (male and female). Lastly, students in each stratum were recruited, using the convenience sampling method until the intended sample size was reached.

### Data collection

The field research team consisted of university graduates in sociology who had been trained in quantitative research methods. The participants were asked to complete the questionnaire anonymously, through the Computer Assisted Self-Interviews (CASI). The structured questionnaire was in the Thai language. It covered the following areas: socio-economic background; recreational activities; smart phone and social media use; intimate relationships; sexual identity and experience; sexually transmitted diseases (STDs); pregnancy, abortion and birth control; and mental health.

### Measurement

#### Primary outcome

The primary outcome was social-psychological well-being (also referred to as psychological well-being in the present manuscript). It was assessed using the Flourishing Scale (FS) [21]. This scale consists of eight items which measure the core aspects of social-psychological functioning, namely purpose and meaning; supportive relationships; engagement; contribution to the well-being of others; competence; self-acceptance; optimism; and being respected. Although the original scale is measured on a seven-point scale, for this study, we used a five-point Likert scale ranging from “Strong disagreement” = 1 to “Strong agreement” = 5. The total score ranges from 8 to 40, with higher scores indicating a person with many psychological resources and strengths. Sample items include “I lead a purposeful and meaningful life” and “I am a good person and live a good life”. The Cronbach’s alpha for this sample was 0.89, indicating a high level of internal consistency.

#### Primary independent variable

“Excessive use of smartphone” was the primary independent variable for this study. We used and adapted five out of the 8 items of the Young Diagnostic Questionnaire for Internet Addiction, since the term “Internet use” in the original scale denotes all types of online activity [25]. In this present study, the term “smartphone” is used to refer, not only to smartphone devices, but also to Internet activities on tablets and old style cell phone. The items were scored as 0 (“No”) or 1 (“Yes”), and consisted of the following: i) you feel preoccupied with your smartphone; ii) You feel the need to use the smartphone with increasing amounts of time in order to achieve satisfaction; iii) You have repeatedly made unsuccessful efforts to control, cut back, or stop smartphone use; iv) You spend time on your smartphone longer than you originally intended; v) You use smartphone as a way of escaping from problems or of relieving a mood problems such as feeling of guilt, sadness, discouragement, unsecure). The total possible score range from 0 to 5. For lack of a clear cut-off level, the score was dichotomized into i) above the median: showing excessive use of smartphones and ii) median and below: not excessive use of smartphones. The observed Cronbach’s alpha in this sample was 0.70.

#### Other covariates

The other covariates were 1) average daily time spent on smartphone; 2) socio-demographic variables (e.g., age, gender; parents’ marital status; parents’ level of education; family household income; perceived financial situation; living situation); 3) variables related to connectedness (frequency of talking with parents; perceived satisfaction with relationships with mother, father, and friends); and 4) gender and sexual related behavior (sexual identity; currently having a boyfriend or girlfriend; and sexual experience).

### Ethics statement

This study received ethical approval from the Human Experimentation Committee at the Research Institute for Health Sciences of Chiang Mai University (Certificate of Ethical Clearance No. 61/2517). The participants were first informed about the objectives of the study; about their roles; about their rights to either give or not give any information during the interviews. They were also informed about the confidentiality of their personal data; and about the way in which the findings of the study would be presented. Participants provided written informed consent prior to participating in the study.

### Statistical analysis

The analysis was performed using SPSS 17 (PASW) for Windows (SPSS Inc., Chicago, Illinois, USA). Categorical independent variables were compared using either the Student’s t-test, ANOVA, or Kruskal-Wallis H test, as determined to be appropriate. Multiple pairwise comparisons were performed using ANOVA with the Bonferroni’s adjustment for those variables which were significant at *p* value < 0.05. Multiple linear regression was performed to primarily assess the correlation of excessive smartphone use (“Yes” versus “No”) with social-psychological well-being (using continuous scores). All the other covariates were systematically included in the model, either because of their potential role as confounders, or for being epidemiologically important. The significance level for the multiple linear regression analysis was set at the *p* value < 0.05. There was no evidence of multicollinearity.

## Results

### Characteristics of participants

Table 1 presents the general characteristics of the participants. Eight hundred students participated in this study; among them, 405 (50.6%) were female, while 395 (49.4%) were male. Slightly over half of the students (52.5%) were 20 years old and above, and the majority (73.4%) had parents who were married or lived together. Fifty-three percent of the students lived in households with monthly incomes between 10,000 and 44,900 Thai Baht (1USD = 35THB at the time of the study), with most of them perceiving that the financial situation of their households was not a problem (62.1%). Two thirds of the students reported talking to parents regularly (62.0%) and were satisfied with their relationships with both parents. An equally high proportion (84.8%) of the students reported being satisfied with their relationships with their friends. The results from multiple pairwise comparisons with Bonferroni’s adjustment are presented in S1 Table.

**Table 1:** Participants’ general characteristics and bivariate analysis with psychological well-being.

	n	%	PWB	
			(mean±SD)	p-value
**Gender**				0.002
Male	395	49.4	32.75±4.87	
Female	405	50.6	33.80±4.88	
**Age**				0.338
≤ 20 years	380	47.5	33.11±5.02	
>20 years	420	52.5	33.44±4.78	
**Education level**				0.444
1^st^ year	188	23.5	33.07±5.06	
2^nd^ - 3^rd^ year	347	43.4	33.16±5.09	
4^th^ - 6^th^ year	265	33.1	33.59±4.51	
**Marital status of parents**				0.197
Divorced/separated	137	17.1	33.12±5.18	
Married/live together	587	73.5	33.20±4.89	
One/both passed away	75	9.4	34.25±4.39	
**Father’s highest level of education**				0.012
Primary education or less	112	14.0	33.04±5.06	
Secondary/high school	201	25.1	33.63±4.86	
College/university	451	56.5	33.39±4.82	
Don’t know	36	4.5	30.78±5.07	
**Mother’s highest level of education**				0.227
Primary education or less	148	18.5	33.30±4.63	
Secondary/high school	207	25.9	33.82±5.18	
College/university	433	54.1	33.06±4.86	
Don’t know	12	1.5	31.92±3.89	
**Household income**				0.768
< 10,000	63	7.9	33.32±5.03	
10,000–44,999	418	52.3	33.37±5.06	
≥ 50,000	294	36.8	33.25±4.52	
Don’t know	25	3.1	32.28±6.14	
**Perceived financial status**				0.489
Financial struggle/“it’s tight”	303	37.9	33.13±5.02	
No financial problems	497	62.1	33.38±4.82	
**Currently live with**				0.007
Family members	368	46.0	33.34±4.86	
Friends	249	31.1	32.60±5.07	
Alone	183	22.9	34.10±4.62	
**Average time spent on using smartphone**				0.182
≤ 2 hours	114	14.2	34.05	
3–4 hours	295	36.9	33.08	
≥ 5 hours	391	48.9	33.21	
**How often do you talk to your parents**				<0.001
Not at all/not often	215	26.9	33.78±4.88	
Neutral	88	11.0	33.92±4.30	
Regularly/often	495	62.0	31.87±4.92	
**Perceived satisfaction with relationship with father**				<0.001
Dissatisfied	32	4.2	30.50±5.80	
Neutral	109	14.4	32.11±4.93	
Satisfied	616	81.4	33.60±4.78	
**Perceived satisfaction with relationship with mother**				<0.001
Dissatisfied	15	1.9	28.53±7.86	
Neutral	65	8.2	31.23±4.64	
Satisfied	711	89.9	33.56±4.78	
**Perceived satisfaction with relation with friends**				<0.001
Dissatisfied	24	3.0	30.08±6.17	
Neutral	98	12.3	31.24±4.59	
Satisfied	678	84.8	33.69±4.78	
**Sexual identity**				0.039
Heterosexual	656	82.0	33.45±4.82	
LBGT	144	18.0	32.52±5.19	
**Currently have a boyfriend/girlfriend**				0.548
No	497	62.1	33.20±4.96	
Yes	303	37.9	33.42±4.80	
**Excessive use of smartphone**				<0.001
No (≤3)	433	54.1	33.99±4.72	
Yes (>3)	366	45.8	32.44±4.98	

PWB: psychological well-being; LGBT: lesbian, gay, bisexual, or transgender

Out of the students, 793 (99.1%) possessed a smartphone, while 729 (91.1%) possessed a tablet, and 76 (9.5%) of the students possessed an old-style cell phone. Nearly half of them (48.9%) spent at least five hours per day on their smartphones. In all, 366 (45.8%) of the students were categorized as being excessive smartphone users. There was a higher proportion of females among the excessive users of smartphones, as compared to students who did not engage in excessive smartphone use behavior (58.7% versus 43.9%; p < 0.001). In addition, more of the excessive smartphone users had parents with overall lower levels of education than the non-excessive users. The trend was similar with income, whereby a lower proportion of excessive smartphone users where came from households with monthly incomes of 10,000 Thai Baht or more (see S2 Table).

### Factors associated with social-psychological well-being

Table 2 displays the correlates of social-psychological well-being among the students. The mean score of psychological well-being for the sample was 33.28 (SD = 4.90) (total score range 8–40). We found that excessive smartphone use was an independent predictor of psychological well-being. The students with excessive levels of smartphone use of had lower scores on the social-psychological well-being than those who did not use smartphone excessively (*B* = -1.60; *P* < 0.001). Female students had psychological well-being scores that were on average 1.24 points higher than scores of male students (*P* < 0.001).

**Table 2:** Multivariable analysis of factors associated with psychological well-being.

		PWB	
	B	95%CI	p-value
**Excessive smartphone use**			
Yes (vs No)	-1.605	-2.313; -0.898	<0.001
**Gender** Female (vs Male)	1.245	0.562; 1.928	<0.001
**Age** in years >20 (vs ≤ 20)	0.015	-0.958; 0.988	0.976
**Education level**			
2^nd^ - 3^rd^ year (vs 1^st^ year)	-0.069	-1.010; 0.872	0.886
4t - 6^th^ year (vs 1^st^ year)	0.178	-1.127; 1.483	0.789
**Marital status of parents**			
Married/Live together (vs Divorced/Separated)	-0.707	-1.665; 0.252	0.148
One/Both passed away (vs Divorced/Separated)	1.208	-0.191; 2.607	0.091
**Father’s highest level of education**			
Primary education or less (ref)			
Secondary/high school	0.573	-0.624; 1.770	0.348
College/university	0.720	-0.574; 2.013	0.275
Don’t know	-2.153	-4.123; -0.183	0.032
**Mother’s highest level of education**			
Primary education or less (ref)			
Secondary/high school	-0.080	-1.167; 1.007	0.885
College/university	-1.028	-2.217; 0.161	0.090
Don’t know	-0.213	-3.178; 2.753	0.888
**Household income**			
10,000–44,999 (vs < 10,000)	-0.128	-1.434; 1.178	0.848
≥ 50,000 (vs < 10,000)	0.039	-1.452; 1.529	0.959
Don’t know (vs < 10,000)	-1.646	-3.901; 0.609	0.152
**Perceived financial status**			
Financial struggle/“it’s tight” (ref)			
No financial problems	-0.219	-0.977; 0.540	0.572
**Currently live with**			
Family members (vs Alone)	-1.053	-1.889; -0.216	0.014
Friends (vs Alone)	-1.521	-2.431; -0.611	0.001
**Time spent using smartphone**			
3-4hours (vs ≤ 2 hours)	-0.686	-1.712; 0.339	0.189
≥ 5 hours (≤ 2 hours)	-0.128	-1.164; 0.907	0.808
**How often do you talk to your parents**			
Regular (vs Not at all/Not often)	0.794	-0.011; 1.598	0.053
Neutral (vs Not at all/Often)	1.531	0.364; 2.699	0.010
**Perceived satisfaction with relation with father**			
Satisfied (vs Dissatisfied)	0.662	-0.703; 2.027	0.341
Neutral (vs Dissatisfied)	0.190	-1.338; 1.718	0.807
**Perceived satisfaction with relation with mother**			
Satisfied (vs Dissatisfied)	1.624	-0.495; 3.743	0.133
Neutral (vs Dssatisfied)	-0.091	-2.444; 2.261	0.939
**Perceived satisfaction with relation with friends**			
Satisfied (vs Dissatisfied)	2.645	0.646; 4.644	0.010
Neutral (vs Dissatisfied)	0.802	-0.343; 2.947	0.463
**LGTB** (vs Heterosexual)	-0.458	-1.320; 0.405	0.298
**Currently have a boyfriend/girlfriend**			
Yes (vs no)	0.443	-0.238; 1.125	0.202

PWB: psychological well-being; LGBT: lesbian, gay, bisexual, or transgender; B: unstandardized regression coefficient; CI: Confidence Interval; ref: reference group

Compared to those students who reported that they do not often talk, or do not talk at all, to their parents, those students who were neutral (*B* = 1.53; *P*< 0.010) and those who reported they regularly or often talk to parents (*B* = 0.79; *P* = 0.053) had higher scores on their psychology well-being. In addition, students who rated the relationship with their friends as satisfactory had on average higher scores on psychological well-being than those were not satisfied (*B* = 2.64; *P* = 0.010). However, perceived satisfaction with relationships with the fathers or mothers was not found to be related to social-psychological well-being. One significant finding, however, was that those students who lived with their family members (*B* = -1.05; *P* = 0.014) or friends (*B* = -1.52; *P* = 0.001) had lower scores of psychological well-being, as compared to students who lived alone.

## Discussion

There is a growing body of evidence showing the association of excessive smartphone use with adverse mental health outcomes such as depression and anxiety [5]. However, there is a remarkable dearth of research which has specifically explored the relationship between smartphone use and psychological well-being. The current study presents evidence linking excessive smartphone use to lower scores of psychological well-being among university students. In this study, we controlled for important socio-economic characteristics and variables related to connectedness to family and friends. This strengthens the results from a previous study that documented a negative correlation between smartphone addiction and psychological well-being, based on a bivariate analysis [23].

The documented relationship between smartphone use and psychological well-being in this study could be bidirectional. The Flourishing Scale used in this study provides an overall assessment of the social-psychological prosperity of individuals. It includes several items on different aspects of social relationships: having supportive and rewarding relationships; contributing to others’s happiness; and being respected by others. It also includes items which cover sense of purpose and meaning in one’s life; being engaged and interested in one’s daily activities; competence; optimism; and self-respect. Based on already existing research, it is possible that smartphone use could interfere with some of these facets of human functioning. For example, Rotondi et al [10], in their recent publication, demonstrated that smartphone use tends to reduce the quality of face-to-face interaction. They found that the positive association between “Time spent with friends” and “Satisfaction with friends” was significantly less strong among individuals who use smartphones. Therefore, individuals who use smartphones excessively, because of their reduced amounts of face-to-face interaction, are likely to have less of a feeling that their social relationships are supportive and rewarding, or less of a feeling that they actively contribute to the happiness and well-being of others. Furthermore, excessive smartphone use is known to often lead to sleep disturbance, which, in turn, tend to affect a person’s mental well-being [26, 27]. Moreover, a growing number of studies among university students have documented high prevalence of nomophobia (abbreviated form of “no mobile phone phobia”). This a new term recently coined to describe the anxieties or feeling of discomfort of losing or being temporarily without one’s mobile phones, and is thought to be related to the excessive use of mobile phones [28–32]. More research is needed to clarify the ways in which smartphone use is likely to affect the different facets of psychological well-being. On the other hand, as suggested by previous studies on depression and anxiety [5, 33, 34], it is likely that those individuals who have lower scores on the Flourishing Scale (indicative of those who do not view themselves in positive terms in important areas of functioning) could resort to excessive smartphone use as a coping strategy to deal with negative emotions.

Nearly half of the participants in this study were classified as excessive users of smartphones. Previous studies among university students in Asia have used various measuring instruments, with prevalence of excessive smartphone use or addiction to smartphone ranging from 19.1% to 36.5% [35–39]. There was no association found between the “Time spent on smartphone” and psychological well-being of the students. Although the “Time spent on smartphone” is an important dimension in assessing problems related to smartphone use, our results suggest that as a single variable “Time spent on smartphone” might not be sensitive enough to capture the effects of problem smartphone use on the psychological well-being of individuals. In this study, being female was associated with higher psychological well-being scores. There are mixed results regarding the association between gender and psychological well-being [40, 41]. Our finding is, however, similar to that reported by Kumcağiz & Gündüz, who used the same scale to measure psychological well-being among university students in Turkey [23].

The quality of relationship with family members and friends is another documented correlate of psychological well-being among students. For example, Daraei [42] found that those students who had lower satisfaction levels of family relationships also had lower scores on their psychological well-being assessment. In the present study, those students who reported engaging in some level of dialogue with their parents (regularly/often and neutral) and those who were satisfied with their relationships with their fathers, mothers, and friends had higher scores on psychological well-being in the bivariate analysis. In the adjusted model, however, only those who reported that they sometimes talked to their parents and those who were satisfied with their relationships with their friends had higher scores on psychological well-being that remained statistically significant. Our findings indicate the importance of connectedness to parents and friends to the psychological well-being of students in Thailand. This is consistent with results of a previous study, which revealed that those youth who reported higher levels of social connectedness at any one point in time would subsequently report higher levels of well-being (i.e., life satisfaction, confidence, positive affect, and aspirations) [43].

However, it is unclear why, in the present study, those students who lived with family members and friends tended to have lower scores on psychological well-being compared to students who lived alone. Previous studies, particularly those conducted in Western societies, have shown that living alone is positively related to life satisfaction and psychological well-being among young adults, since living alone can increase feelings of self-sufficiency and independence, which are critical developmental tasks [44], and are qualities which are highly valued in most Western cultures. Therefore, it is possible that, in this study, the higher psychological well-being scores among students who lived alone in this study could be explained by life stage, as well as by the increasing influence of Western lifestyles and attitude. Living alone does not necessarily relate to lack of social support, since students can still maintain close contact with their family of origin and have active social networks of friends [45].

The results of this study should be interpreted in light of its limitation. Firstly, this study is correlational in design; thus, no causal associations can be inferred. Secondly, our assessment of smartphone use consisted of only five items, which were adapted from the eight items of the Young Diagnostic Questionnaire for Internet Addiction. Although the term “Internet use”, as written in the original scale, denotes all types of online activity, problem smartphone use is likely to have additional features which are specifically related to the nature of smartphones, which might not reflected on the Young Diagnostic Questionnaire for Internet Addiction. In addition, because we included only five items out of the original eight from the Young Diagnostic Questionnaire for Internet Addiction, it was not possible to use the categorization scheme of the original scale. The use of the median as a cutoff point could have also resulted in the loss of some information. Lastly, it is important to note that one significant limitation to the present study relates to the fact that our measurements of the social-psychological well-being and of excessive smartphone use have not yet been validated within the context of Thailand. Nonetheless, this study is one of the rare available studies which has documented the relationship between smartphone use and psychological well-being among university students.

In conclusion, our results have provided the first insights into the negative association of excessive smartphone use with the psychological well-being among university students in Thailand. Although more research is needed to corroborate our findings, our results suggest the critical need to promote healthy ways of smartphone use, as well as the importance of friendship and family connectedness as a way of promoting the psychological well-being of students.

## Supporting information

S1 Dataset Dataset of the study. (SAV)

S1 Table Pairwise comparisons: Bonferroni tests for differences in mean of psychological well being scores. (DOCX)

S2 Table Socio-demographic characteristics of excessive and non-excessive smartphone users. (DOCX)
