# Supplementary material for: Association of excessive smartphone use with psychological well-being among university students in Chiang Mai, Thailand
Source: PLoS One. 2019 Jan 7;14(1):e0210294. doi: 10.1371/journal.pone.0210294 (PMC6322718; doi:10.1371/journal.pone.0210294)
Supplement: S1 Table — (DOCX) [file pone.0210294.s002.docx]

**S1 Table**. Pairwise comparisons: Bonferroni tests for differences in mean of psychological well being scores

|  | **Psychological well-being** | | |
| --- | --- | --- | --- |
|  | Mean difference | CI | p-value |
| **Father’s highest level of education** |  |  |  |
| Primary education or less vs Secondary/high school | -0.587 | -2.11; 0.93 | 1.000 |
| Primary education vs college/university | -0.341 | -1.70; 1.02 | 1.00 |
| Primary education vs don’t know | 2.267 | -0.20; 4.74 | 0.093 |
| Secondary/high school vs college/university | 0.246 | -0.93; 2.11 | 1.000 |
| Secondary/high school vs don’t know | 2.854 | 0.52; 5.19 | 0.008 |
| college/university vs don’t know | 2.608 | 0.37; 4.84 | 0.013 |
| **Currently live with** |  |  |  |
| Family members vs friends | 0.735 | -0.23; 1.69 | 0.201 |
| Family members vs alone | -0.761 | -1.82; 0.30 | 0.254 |
| Friends vs alone | -1.496 | -2.64; -0.36 | 0.005 |
| **How often do you talk to your parents** |  |  |  |
| Not at all/not often vs neutral | -2.046 | -3.51; -0.58 | 0.003 |
| Not at all/not often vs Regularly/often | -1.901 | -2.85; -0.95 | <0.001 |
| Regularly/often vs neutral | -0.145 | -1.49; 1.20 | 1.000 |
| **Perceived satisfaction with relationship with father** |  |  |  |
| Dissatisfied vs satisfied | -3.104 | -5.22; -0.99 | 0.001 |
| Dissatisfied vs Neutral | -1.610 | -3.95; 0.73 | 0.299 |
| Satisfied vs Neutral | 1.494 | 0.28; 2.70 | 0.010 |
| **Perceived satisfaction with relationship with mother** |  |  |  |
| Dissatisfied vs satisfied | -5.029 | -8.06; -2.00 | <0.001 |
| Dissatisfied vs Neutral | -2.607 | -6.03; 0.63 | 0.157 |
| Satisfied vs Neutral | 2.332 | 0.83; 3.84 | 0.001 |
| **Perceived satisfaction with relation with friend** |  |  |  |
| Dissatisfied vs satisfied | -3.607 | -6.00; -1.21 | 0.001 |
| Dissatisfied vs Neutral | -1.162 | -3.79; 1.47 | 0.868 |
| Satisfied vs Neutral | 2.445 | 1.20; 3.69 | <0.001 |

CI: confidence Interval; vs: versus
