# Supplementary material for: Association of excessive smartphone use with psychological well-being among university students in Chiang Mai, Thailand
Source: PLoS One. 2019 Jan 7;14(1):e0210294. doi: 10.1371/journal.pone.0210294 (PMC6322718; doi:10.1371/journal.pone.0210294)
Supplement: S2 Table — (DOCX) [file pone.0210294.s003.docx]

**S2 Table**. Socio-demographic characteristics of excessive and non-excessive smartphone users

|  |  | **Excessive smartphone use** | | **p-value** |
| --- | --- | --- | --- | --- |
|  |  | **Yes** | **No** |  |
|  | n (%) | n (%) | n (%) |  |
| **Gender** |  |  |  | <0.001 |
| Male | 394 (49.3) | 151 (41.3) | 243 (56.1) |  |
| Female | 405 (50.7) | 215 (58.7) | 190 (43.9) |  |
| **Age** |  |  |  | 0.324 |
| ≤ 20 years | 380 (47.6) | 199 (46.0) | 181 (49.5) |  |
| >20 years | 419 (52.4) | 234 (54.0) | 185 (50.5) |  |
| **Education level** |  |  |  | 0.108 |
| 1^st^ year | 188 (23.5) | 92 (25.1) | 96 (22.2) |  |
| 2-3^rd^ year | 347 (43.4) | 167 (45.6) | 180 (41.6) |  |
| 4-6^th^ year | 264 (33.0) | 107 (29.2) | 157 (36.3) |  |
| **Marital status of parents** |  |  |  | 0.654 |
| Divorced/separated | 137 (17.2) | 63 (17.3) | 74 (17.1) |  |
| Married/live together | 586 (73.4) | 264 (72.3) | 322 (74.4) |  |
| One/both passed away | 75 (9.4) | 38 (10.4) | 37 (8.5) |  |
| **Father’s highest level of education** |  |  |  | <0.001 |
| Primary education or less | 112 (14.0) | 71 (19.4) | 41 (9.5) |  |
| Secondary/high school | 200 (25.0) | 94 (25.7) | 106 (24.5) |  |
| College/university | 451 (56.4) | 185 (50.5) | 266 (61.4) |  |
| Don’t know | 36 (4.5) | 16 (4.4) | 20 (4.6) |  |
| **Mother’s highest level of education** |  |  |  | 0.012 |
| Primary education or less | 148 (18.5) | 81 (22.1) | 67 (15.5) |  |
| Secondary/high school | 206 (25.8) | 103 (28.1) | 103 (23.8) |  |
| College/university | 433 (54.2) | 176 (48.1) | 257 (59.4) |  |
| Don’t know | 12 (1.5) | 6 (1.6) | 6 (1.4) |  |
| **Household income** |  |  |  | 0.037 |
| < 10,000 | 62 (7.8) | 36 (9.8) | 26 (6.0) |  |
| 10,000-44,999 | 418 (52.3) | 201 (54.9) | 217 (50.1) |  |
| ≥ 50,000 | 294 (36.8) | 118 (32.2) | 176 (40.6) |  |
| Don’t know | 25 (3.1) | 11 (3.0) | 14 (3.2) |  |
| **Perceived financial status** |  |  |  | 0.008 |
| Financial struggle/it’s tight | 303 (37.9) | 157 (42.9) | 146 (33.7) |  |
| No financial problem | 496 (62.1) | 209 (57.1) | 287 (66.3) |  |
| **Currently live with** |  |  |  | 0.383 |
| Family members | 367 (45.9) | 161 (44.0) | 206 (47.6) |  |
| Friends | 249 (31.2) | 123 (33.6) | 126 (29.1) |  |
| Alone | 183 (22.9) | 82 (22.4) | 101 (23.3) |  |
| **Average time spent on using smartphone** |  |  |  | <0.001 |
| ≤ 2 hours | 114 (14.3) | 17 (4.6) | 97 (22.4) |  |
| 3-4 hours | 294 (36.8) | 110 (30.1) | 184 (42.5) |  |
| ≥ 5 hours | 391 (48.9) | 239 (65.3) | 152 (35.1) |  |
